# Measurement of burnout during the prolonged pandemic in the Chinese zero-COVID context: COVID-19 burnout views scale

**DOI:** 10.3389/fpubh.2022.1039450

**Published:** 2022-11-10

**Authors:** Sam S. S. Lau, Cherry C. Y. Ho, Rebecca C. K. Pang, Susan Su, Heather Kwok, Sai-fu Fung, Roger C. Ho

**Affiliations:** ^1^Research Centre for Environment and Human Health, School of Continuing Education, Hong Kong Baptist University, Hong Kong, China; ^2^Multidisciplinary Research Centre, School of Continuing Education, Hong Kong Baptist University, Hong Kong, China; ^3^College of International Education, Hong Kong Baptist University, Hong Kong, China; ^4^Institute of Bioresources and Agriculture, Hong Kong Baptist University, Hong Kong, China; ^5^Division of Nursing Education, School of Continuing Education, Hong Kong Baptist University, Hong Kong, China; ^6^Department of Social and Behavioural Sciences, City University of Hong Kong, Kowloon, Hong Kong SAR, China; ^7^Department of Psychological Medicine, National University of Singapore, Singapore, Singapore

**Keywords:** measure, burnout, instrument development, psychometric testing, Chinese, validation

## Abstract

Burnout is an important public health issue at times of the COVID-19 pandemic. Current measures which focus on work-based burnout have limitations in length and/or relevance. When stepping into the post-pandemic as a new Norm Era, the burnout scale for the general population is urgently needed to fill the gap. This study aimed to develop a COVID-19 Burnout Views Scale (COVID-19 BVS) to measure burnout views of the general public in a Chinese context and examine its psychometric properties. A multiphase approach including literature review, expert consultation, and pilot testing was adopted in developing the scale. The scale was administered to a sample of 1,078 of the general public in Hong Kong with an average age of 34.45 years (SD = 12.47). Exploratory and Confirmatory Factor Analyses suggested a 5-item unidimensional model of COVID-19 BVS. The CFA results indicated that the COVID-19 BVS had a good model fit, as χ2 (10.054)/5 = 2.01, SRM*R* = 0.010, CFI = 0.998, RMSEA = 0.031. Five items were maintained in EFA with high internal consistency in terms of Cronbach's α of 0.845 and McDonald's ω coefficient of 0.87, and the corrected item-to-total correlations of 0.512 to 0.789 are way above the acceptable range. The KMO values of 0.841 and Bartlett's Test of Sphericity (*p* < 0.01) verified the normal distribution of the EFA and the adequacy of the EFA sampling. The analyses suggest that the COVID-19 BVS is a promising tool for assessing burnout views on the impacts of the epidemic on the Chinese general populations.

## Background

The COVID-19 crisis has become one of the most important public health concerns in many countries. As of 7 September 2022, 603 million confirmed cases including 6.48 million of deaths pertaining to COVID-19 have been reported globally (1). In the first quarter of 2022, Hong Kong was amongst the hardest hit as it embraced the COVID's fifth wave caused by the variant Omicron. It brought about a significant increase in the number of confirmed cases hitting 5-digit cases daily. To respond to the rapid spread of COVID, in alignment with China's zero-COVID strategy adopting among the strictest approaches to prevent and control the pandemic anywhere in the world, the Hong Kong Government has adopted “dynamic zero-COVID” policy and implemented a number of stringent anti-pandemic measures, such as social distancing, contact tracing, dine-in ban, suspension of face-to-face teaching and learning, adoption of working from home, compulsory testing and quarantine measures, and travel restriction.

The prolonged anti-pandemic measures have been causing significant disruption to people's daily routine such as lack of social interactions, feeling restricted, shifted to work from home, being laid off, and resulted in financial difficulties, which may deteriorate mental health (2–4). In addition to the well documented mental health impacts on the healthcare professionals (5), increasing research has revealed that COVID-19 has significantly affected individuals' mental health causing anxiety (6), stress, fears (7), mental confusion, social deprivation, depression (8), panic (9), psychological burnout (10) in the general population. A recent study showed that COVID-19 could develop symptoms of Post-Traumatic Stress Disorder (PTSD) (11). In the United States, it is estimated that around 40% of the general population suffered from psychological distress due to the COVID-19 pandemic, and 69% of them felt burnout while working from home (12). In China, where strict restriction measures are adopted to align with the Zero-COVID strategy, Qiu et al. (13) reported around 35% of the 52,370 study participants experienced psychological distress. Chen et al. (14) revealed that anxiety and depression were common in the general public during the pandemic. In Hong Kong, the persistent uncertainties brought by COVID-19 regarding the spread of the virus, the unpredictable future, repeated waves with variants, the prolonged period of implementing strict anti-epidemic measures, continuous changes of plans and social gatherings, fear of being infected and quarantine for an extended period, changes in daily routines, and streaming of COVID-related news may lead people are subject to exhaustion and burnout (2, 15, 16). The government has recently recognized people's increasing intolerance in adopting the prolonged health-protective measures, leading to “pandemic burnout” (10).

The concept of Burnout was initially identified in the workplace context (17), and now it is expanded to other situations, such as chronic stressors (18). The core symptoms of work-based burnout are overwhelming exhaustion, feelings of cynicism and detachment from the job, a sense of ineffectiveness, and lack of accomplishment [cited from 10]. “Burnout” is defined as a psychological syndrome characterized by emotional exhaustion, feelings of cynicism and reduced personal accomplishment (19). It has been found that burnout caused by a pandemic can make people feel emotionally drained and affect people's every aspect of their life (20). People who are stuck in pandemic burnout may lead to lower levels of motivation, feelings of helplessness, loneliness, hopelessness, depersonalization, reduced personal achievement (21), and non-compliance with health protective measures as the pandemic continues without certainty of the pandemic's end, and the changing social distancing measures, not being able to socialize with other people, being restricted on freedom of movement, feel despair (22). In this connection, people attitude to COVID may be getting more embracing, and start to loosen the public health measures to halt the transmission of virus (20, 22). Therefore, dealing with pandemic burnout is of utmost importance as if this psychological problem is left unattended, the spread of COVID-19 will be uncontrolled, causing more deaths and negative impacts on the public.

Currently, majority of the research examined pandemic burnout focused on physicians, nurses and educators [e.g., (7, 23, 24)], with only limited studies focused on the general public (7) and rarely addressed COVID-related burnout in relation to zero-COVID policy [e.g., (25)]. It has been demonstrated that high levels of burnout are significantly associated with psychological burden, depression, anxiety and insomnia (7). To assess the level of burnout of the general population, and plan and develop care for the general population to fight the prolonged COVID-19 crisis, a valid and reliable assessment tool is urgently needed to identify the factors associated with burnout during the pandemic and the consequences of burnout on individuals. To address this need, the study aimed to develop the COVID-19 Burnout Views Scale (COVID-19 BVS) and evaluate its psychometric properties.

## Methods

### Setting

Being part of the larger project “COVID-related experience and psychological impacts during the Omicron-dominant COVID-19 Pandemic”, this study was a methodological study using classical test theory in psychometric analysis. The ethics approval was obtained from Hong Kong Baptist University's Research Ethics Committee (REC/21-22/0353). The anonymity of the participants was maintained.

### Procedures and participants

Data collection took place between 8 and 27 March 2022 in Hong Kong, China during which the city was subject to surging confirmed cases and death of the 5th pandemic wave driven by the Omicron variant. A snowball sampling strategy was adopted, focusing on recruiting the general public. An online self-administered survey on the Qualtrics platform was circulated *via* emails, and social media networks with linked QR codes (that can be read using a digital device). Before starting the survey, participants were informed about the consent of the survey and asked for their consent. Inclusion criteria were as follows: (a) Age ≥ 18 years; (b) Ability to read and understand Chinese (since the survey was conducted in Chinese only); (c) Living in Hong Kong at the time of the survey; (d) Accessibility to internet as the study adopted an online survey; and (e) Provision of an informed consent to participate. Participation in the study was on a voluntary basis, no compensation was provided to the participant. The valid dataset was completed by 1,078 eligible participants with an average age of 34.45 years (SD = 12.47), 72.8% of the participants were women (*n* = 785). In the sample, 9% participants (*n* = 96) reported having a chronic disease, 97.03% of the participants (*n* = 1,045) reported being vaccinated.

### Pilot study for developing the first version of COVID-19 Burnout Views Scale

#### Development of the COVID-19 Burnout Views Scale (COVID-19 BVS)

**Figure 1:**
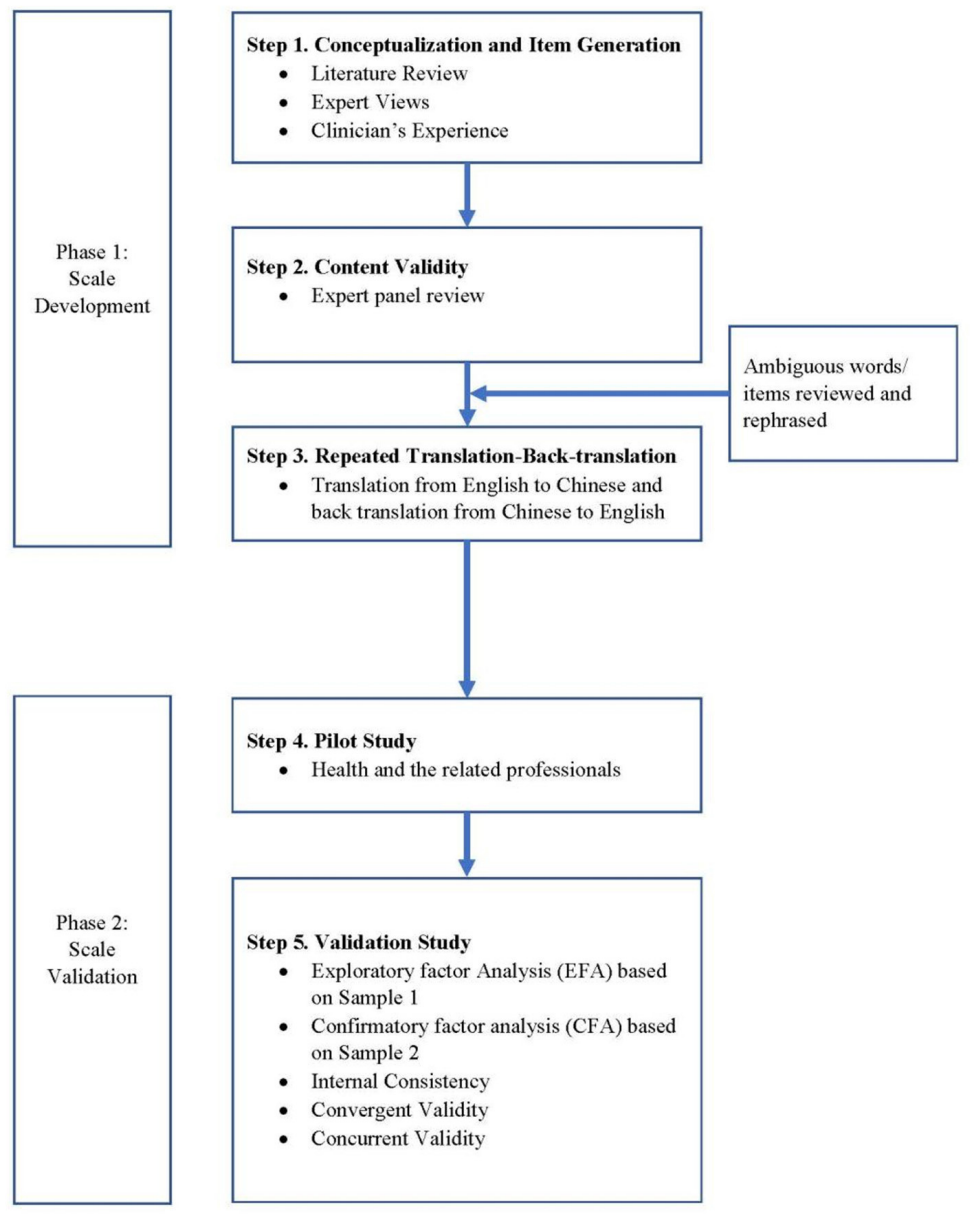
A flow chart depicting the steps involved in the development and validation of the scale.

The COVID-19 BVS was developed for assessing views of burnout encountered by the general population. The “pandemic fatigue” coined by the WHO provided the conceptual foundation for the items in the COVID-19 BVS (20). The WHO defines “*pandemic fatigue” as demotivation to follow recommended protective behaviours, emerging gradually over time and affected by a number of emotions, experiences and perceptions”*. A multiphase approach was adopted in the development of the COVID-19 BVS (Figure 1). To develop the scale, first, a literature review was conducted to identify existing questionnaires pertaining to the measurement of burnout by health care workers before the COVID-19 pandemic (26, 27) and during the COVID-19 crisis (28), with 7 items developed with respect to the COVID-19 context. Second, to ensure that content validity is evaluated adequately, experts in health-related professionals were selected to form an expert panel to review and edit the questions. The experts included medical doctor, psychologist, nursing professional, health researcher, social scientist, and social worker. Third, the repeated forward-backward translation procedure was adopted as it is most commonly quoted in the adaptation and translation process (29). The forward and backward translation procedures were repeatedly adopted. The Chinese version was revised accordingly, and again back-translated. This forward-backward process was repeated until satisfactory agreement was reached. Fourth, during a pilot test with 27 individuals from the different professionals (e.g. in sports, medical fields, healthcare sectors, science), wordings of the items were tested in order to make reading and comprehension accessible. The final version was read and approved by the first three authors together. The 7-item COVID-19 BVS was developed in assessing the views on burnout on a seven-point Likert scale, with 1 indicating Strongly disagree and 7 indicating Strongly agree. The higher score represented a higher level of burnout. The 7 questions covered emotional exhaustion (1 question), loss of interest (1 question), views on personal accomplishment (1 question), perspective, adherence and appropriateness of COVID-19 prevention strategies (3 questions) as well as perceived effectiveness measures to reduce burnout (1 question).

### Statistical analysis

There were several steps involved in the validation of the COVID-19 BVS. To begin with, the entire dataset (*N* = 1,078) was randomly stratified into two datasets (Sample 1, *n* = 539; Sample 2, *n* = 539) to avoid computing the EFA and CFA on the same dataset to prevent overfitting (30–32). Sample 1 (*n* = 539) was then used to shortlist the items for the COVID-19 BVS using a stepwise confirmatory factor analytical approach (SCOFA) (33) together with the standard scale development procedure using exploratory factor analysis (EFA) using principal axis factoring method (34, 35). The larger the KMO coefficient, the more suitable for factor analysis. Model fit was evaluated using KMO coefficient and Bartlett's tests, with values >0.70 and *p* < 0.01, respectively, indicating good fit (36). The evaluated items had a factor loading of higher than 0.50 and factor possessed an eigenvalue higher than 1.0 (37).

Subsequently, confirmatory factor analysis (CFA) was employed to further verify the construct validity of the COVID-19 BVS based on Sample 2 (*n* = 539) (36). With reference to the recent simulation studies on the selection of CFA estimator, weighted least square mean and variance adjusted estimators (WLSMV) was used as the estimation method (38, 39). We used several fit indices (and the respective cutoff criteria) to determine model fit: CFI > 0.950, RMSEA < 0.05, SRMR < 0.05 (40–43) as well as the χ^2^/df ≤ 3 (44, 45). We used Cronbach's α reliability coefficient and Cronbach's α if-item-deleted (46), the McDonald's ω coefficient (47), and the corrected item-total correlation between the five items (37, 48) to assess internal consistency of the COVID-19 BVS.

In terms of convergent and concurrent validity, we evaluated them using the pattern of Spearman correlations with Fear of COVID-19 (FCV-19S) and other construal-related scales, respectively. The 7-item FCV-19S was developed by Ahorsu et al. (49) to assess fear toward COVID-19, where higher score is indicative of more fear toward COVID-19. This scale showed good internal validity (α = 0.82) and was applied to analyse the convergent validity of the COVID-19 BVS. Other construal-related scales, including age, presence of chronic illnesses, support for living with the COVID policy, status of COVID-19 vaccination, and attitude about dynamic Zero COVID-19 strategy were used to analyse the concurrent validity of the COVID-19 BVS. Based on the current research findings, we expected a positive relationship of COVID-19 BVS with (i) FCV-19S (49), (ii) presence of chronic illnesses (50), and (iii) support for living with the pandemic, i.e., by reducing the social distancing measures (51). Research shows that burnout level may influence people's inertia or inaction toward vaccination (52). A negative association between the COVID-19 BVS and the status of COVID-19 vaccination (53) was expected. With reference to the literature, we also expected the COVID-19 BVS to be negatively associated with age (50), and attitude about dynamic Zero COVID-19 strategy (41, 54).

SPSS version 26.0, Mplus version 8.5 and psych package in R computing environment (4.1.1) were used of the analyses.

## Result

### Factorial validity and structure

**Figure 2:**
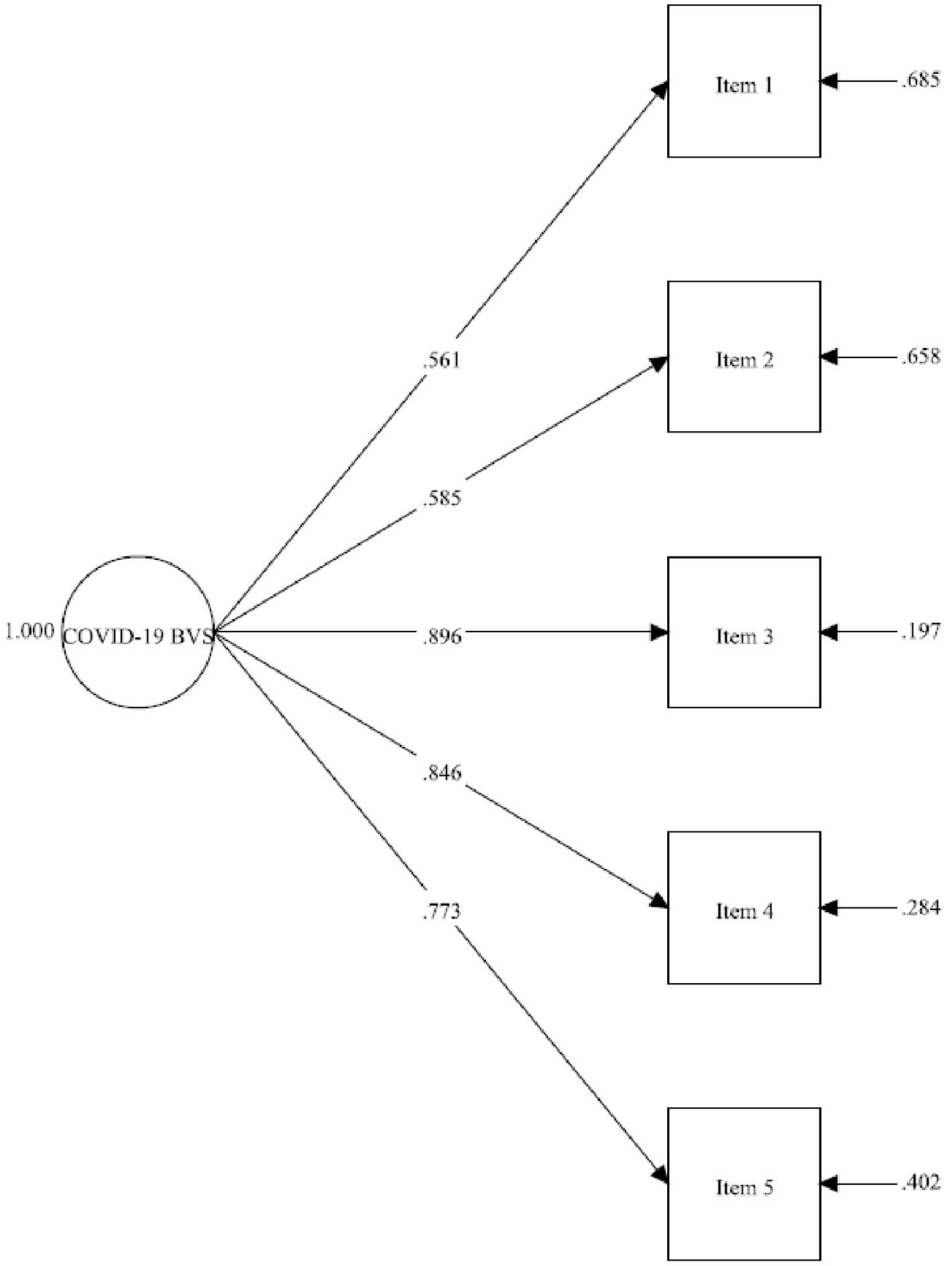
Estimated model of the 5-item COVID-19 Burnout Views Scale (Sample 2, *n* = 539).

The items for constructing the COVID-19 BVS were selected using SCOFA (32). The two items with unsatisfactory factor loading were removed, including: ‘There is a loss of clear perspective on my work, study or life during the COVID-19 pandemic'; and ‘I will move to other cities or countries that have less restrictions COVID-19'. The remaining 5 items that were further verified with reference to the exploratory factor analysis (EFA) results. As shown in Table 1, the results of EFA using principal axis factoring method from Sample 1 (*n* = 539) showed that the 5-item unidimensional factor structure (eigenvalue value = 3.069) is meritorious for the COVID-19 BVS, with a KMO value of 0.841 (χ^2^ = 1097.353; df = 10, *p* < 0.001), with factor loadings ranging from 0.525 to 0.889, and with the overall explanatory power of 53.040%. To further evaluate the construct validity of the COVID-19 BVS, CFA was computed on the Sample 2 (*n* = 539). As shown in Figure 2, the model fit indices were shown to be good, with χ2 (6.899)/5 = 1.38, SRM*R* = 0.012, CFI = 0.998, RMSEA = 0.027 [90% CI 0.000–0.070], with AVE and CR values 0.656 and 0.858, respectively. We also analyzed the entire data set (*N* = 1,078) with the CFA, the results in general replicated the sub-sample's findings, as χ^2^ (10.054)/5 = 2.01, SRM*R* = 0.010, CFI = 0.998, RMSEA = 0.031 [90% CI 0.000–0.058]. The standardized estimate for the items is ranged from 0.559 to 0.891. The results show that the 5-item COVID-19 BVS has achieved all the cutoff criteria of good model fit indices for a unidimensional factor structure without any *post hoc* modifications.

**Table 1:** EFA with principal axis factoring method (Sample 1, *n* = 539).

**Item**		**Sample 1 (*n =* 539)**
1.	I'm not interested in what is going on with the COVID-19 pandemic and the preventive measures implemented by the government.	0.608
2.	I accomplish less and less in my life since the COVID-19 pandemic.	0.525
3.	I find the current COVID-19 preventive measures are disproportionate and worsen my burnout.	0.889
4.	I want to reduce my adherence to COVID-19 preventive measures to avoid burnout.	0.784
5.	The COVID-19 preventive measures are ineffective and the pandemic situation is worsened by burnout.	0.775

### Internal consistency

Table 2 presents the descriptive statistics based on the entire data set (*N* = 1,078). The Cronbach's α of 0.845, McDonald's ω coefficient of 0.87, and the item-to-total correlations for the COVID-19 BVS of a range from 0.512 to 0.789 are way above the acceptable range, indicating a good internal consistency of the scale.

**Table 2:** Descriptive statistics for the COVID-19 Burnout Views Scale.

**Item**	**x**	**SD**	**sk**	**ku**	**rit**	**aiid**
COVID-19 Burnout Views Scale	23.67	6.892	−0.461	−0.262	–	–
Item 1	4.07	1.779	0.085	−1.062	0.547	0.843
Item 2	5.15	1.588	−0.846	−0.073	0.512	0.849
Item 3	5.13	1.723	−0.827	−0.248	0.789	0.777
Item 4	4.49	1.892	−0.323	−1.062	0.728	0.793
Item 5	4.84	1.770	−0.554	−0.685	0.702	0.801

sk, Skewness; ku, Kurtosis; r_it_, Corrected item-total correlations; a_iid_, Cronbach's alpha, if item deleted.

### Convergent and concurrent validity

Table 3 shows Spearman's correlation coefficients between the COVID-19 BVS with the FCV-19S for testing convergent validity and with other construct-related measures for testing concurrent validity, using the entire data set (*N* = 1,078). The results show a significant weak positive correlation between the scale and chronic illness history, with 0.094 and 0.095 (using point-biserial correlation analysis), both are statistically significant at *p* < 0.001. There is significant moderate to strong positive relationship of the scale with the following statements: ‘I support the government to adopt the “living with the COVID” policy instead of the “Dynamic Zero COVID-19 strategy” (*r* = 0.504, *p* < 0.001) and ‘The “Dynamic Zero COVID-19 strategy” is not sustainable in the long run' (*r* = 0.521, *p* < 0.001). The results showed a significant negative relationships of the COVID-19 Burnout Views Scale with Fear of COVID-19 Scale (*r* = −0.129 (*p* < 0.001), COVID-19 vaccination status (*r* = −0.285, *p* < 0.001), age (*r* = −0.241, *p* < 0.001), perceiving the “Dynamic Zero COVID-19 strategy” as an effective measure (*r* = −0.546, *p* < 0.001) and supporting the “Dynamic Zero COVID-19 strategy” as a long term goal (*r* = −0.576, *p* < 0.001).

**Table 3:** Correlations between the COVID-19 Burnout Views Scale in relation to Fear of COVID-19 Scale and other construct-related measures.

**Scale**	**COVID-19 BVS Views Scale**
Do you have any chronic illness (e.g. diabetes, kidney problem, cancer)?	0.094***
I support the government to adopt the “living with the COVID” policy instead of the “Dynamic Zero COVID-19 strategy”.	0.504***
The “Dynamic Zero COVID-19 strategy” is not sustainable in the long run.	0.521***
Fear of COVID-19 Scale	−0.129***
Age	−0.285***
COVID-19 vaccination status	−0.241***
“Dynamic Zero COVID-19 strategy” is an effective measure to protect my city against COVID-19.	−0.546***
I support the “Dynamic Zero COVID-19 strategy” to continue and remain the ultimate goal in the long run.	−0.576***

***p < 0.001.

## Discussion

Aligning with China, the dynamic zero-COVID strategy adopted by Hong Kong, China, which is one of the stringent anti-pandemic measures in the world, has been leading to exhaustion and burnout of the general population. The exhaustion and burnout would have been further exacerbated due to the fast-spreading variant omicron hitting Hong Kong since March 2022, leading to significant impacts on the living and health-protective experience of the Hong Kong people. The aim of the study was to develop a scale and assess its validity and reliability so that researchers and health workers can use it to assess the levels of burnout views associated with the prolonged COVID-19 pandemic with zero-COVID measures. To this end, we examined the psychometric properties of the COVID-19 BVS with a large Hong Kong sample.

The present study developed a 5-item COVID-19 BVS which was shown to be a sound psychometric tool for measuring burnout views, with good construct, convergent and concurrent validity and high internal consistency. As expected, our results revealed that the COVID-19 BVS demonstrated the convergent and concurrent validity. Our analyses indicated that the COVID-19 BVS was negatively correlated with Fear of COVID-19 Scale, which is not consistent with the literature (49). Ahorsu et al. (55) suggested that burnout served as the mediator with fear of COVID-19 being found to have a significant direct effect on burnout. Other studies also revealed a positive effect of fear of COVID-19 on stress and a prolonged exposure to stress would result in burnout (56–58). In line with other studies, we found the expected positive associations of the COVID-19 BVS with the presence of chronic illnesses (50), support for “living with the COVID” policy, i.e., by reducing the social distancing measures (51). Congruent with the literature, we found expected negative association of COVID-19 with age (13, 50, 54, 59), status of COVID-19 vaccination (53), and attitude toward “dynamic Zero COVID-19 strategy” (41, 54).

There are several shortcomings in this study which should be noted. First, the samples in the pilot and main studies are not probability samples and are therefore not truly representative of the general population in Hong Kong. For instance, the higher percentage of female than male in the samples may reflect that female was more likely to volunteer for research [e.g. (59)]. Second, because participation was voluntary, it is possible that the study participants showed selective attention to this study topic. It can be assumed that we were only able to recruit motivated individuals, but those who did not participate might have had different experiences. Third, test-retest reliability of the scale was not conducted. Fourth, further studies to verify the concurrent and discriminant validity of the scale with mental health and well-being scales are warranted. Despite the shortcomings, this study has a notable strength, that is, we used a large sample of data, which allowed us to split the overall sample into two subsamples, and then perform one EFA and one CFA; and the scale was systematically tested for validity and reliability. These strengthened the evidence for the proposed unidimensional 5-item model of COVID-19 BVS.

In future studies, researchers are encouraged to replicate this study with random, representative samples. Future validation research is needed especially with prospective longitudinal studies to examine the predictive validity of the COVID-19 BVS. Further research should attempt to study the association between COVID-19 burnout and post COVID syndrome in people who have recovered from COVID-19 (60). Cross-cultural validation of COVID-19 BVS is important as different cultures have specific factors associated with burnout and negative mental health (e.g., prolonged face mask use) (61). Additional research is also needed to examine the relationship between pandemic burnout views and fear of COVID-19.

## Implications

In view of the increasing attention and concerns surrounding pandemic-related burnout, the current research contributes to the burnout literature by developing the COVID-19 BVS. To the authors' knowledge, it is amongst the first studies in the burnout literature that concerns the burnout views in the context of zero-COVID strategy. Lau et al. (25) developed the COVID-19 Burnout Frequency Scale to measure the COVID-19 burnout in terms of frequency in the context of zero-COVID strategy. Given the burnout risks that are associated with the prolonged COVID-19 pandemic—and with the lesser attention given to the general population due to the dynamic zero-COVID strategy—burnout of the Chinese is worthy of future study. Practically, the COVID-19 BVS provides a brief, valid instrument and appropriate for monitoring Chinese burnout as the COVID-19 crisis and the zero-COVID strategy continue.

## Conclusions

The current research developed a new instrument for evaluating burnout of Chinese in Hong Kong. The 5-item COVID-19 BVS was demonstrated to have good psychometric properties with construct, convergent and concurrent validity evident and high reliability.

## Data availability statement

The raw data supporting the conclusions of this article will be made available by the authors, without undue reservation.

## Ethics statement

The studies involving human participants were reviewed and approved by Hong Kong Baptist University's Research Ethics Committee. The patients/participants provided their written informed consent to participate in this study.

## Author contributions

SL and RH conceptualized and designed the methodology of the study. CH, RP, and SS assisted SL and RH in the development of methodology. SL, CH, RP, SS, and HK performed the data collection. SL, S-fF, CH, and RP conducted statistical analysis. SL, S-fF, CH, RP, and RH drafted and wrote the manuscript. SL oversaw the project administration and supervision and acquired financial support. All authors contributed to the article and approved the submitted version.

## Funding

This study was conducted with the support of a grant from the RGC of the HKSAR, China (UGC/IDS(R) 23/20).

## Conflict of interest

The authors declare that the research was conducted in the absence of any commercial or financial relationships that could be construed as a potential conflict of interest.

## Publisher's note

All claims expressed in this article are solely those of the authors and do not necessarily represent those of their affiliated organizations, or those of the publisher, the editors and the reviewers. Any product that may be evaluated in this article, or claim that may be made by its manufacturer, is not guaranteed or endorsed by the publisher.
